# Inverse design of color routers in CMOS image sensors: toward minimizing interpixel crosstalk

**DOI:** 10.1515/nanoph-2024-0269

**Published:** 2024-07-01

**Authors:** Sangbin Lee, Jaehyun Hong, Joonho Kang, Junjeong Park, Jaesung Lim, Taeho Lee, Min Seok Jang, Haejun Chung

**Affiliations:** Department of Artificial Intelligence Semiconductor Engineering, 26716 Hanyang University, Seoul, 04763, South Korea; Department of Electronic Engineering, 26716 Hanyang University, Seoul, 04763, South Korea; Department of Physics, 26716 Hanyang University, Seoul, 04763, South Korea; School of Electrical Engineering, Korea Advanced Institute of Science and Technology, Daejeon, 34141, Republic of Korea; Department of Electronic Engineering and Department of Artificial Intelligence and Department of Artificial Intelligence Semiconductor Engineering, 26716 Hanyang University, Seoul, 04763, South Korea

**Keywords:** inverse design, color routing, image sensor, adjoint optimization, crosstalk

## Abstract

Over the past decade, significant advancements in high-resolution imaging technology have been driven by the miniaturization of pixels within image sensors. However, this reduction in pixel size to submicrometer dimensions has led to decreased efficiency in color filters and microlens arrays. The development of color routers that operate at visible wavelengths presents a promising avenue for further miniaturization. Despite this, existing color routers often encounter severe interpixel crosstalk, around 70 %, due to the reliance on periodic boundary conditions. Here, we present interpixel crosstalk-minimized color routers that achieve an unprecedented in-pixel optical efficiency of 87.2 % and significantly reduce interpixel crosstalk to 2.6 %. The color routers are designed through adjoint optimization, incorporating customized incident waves to minimize interpixel crosstalks. Our findings suggest that our color router design surpasses existing color routing techniques in terms of in-pixel optical efficiency, representing a crucial step forward in the push toward commercializing the next generation of solid-state image sensors.

## 1 Introduction

**Figure 1:**
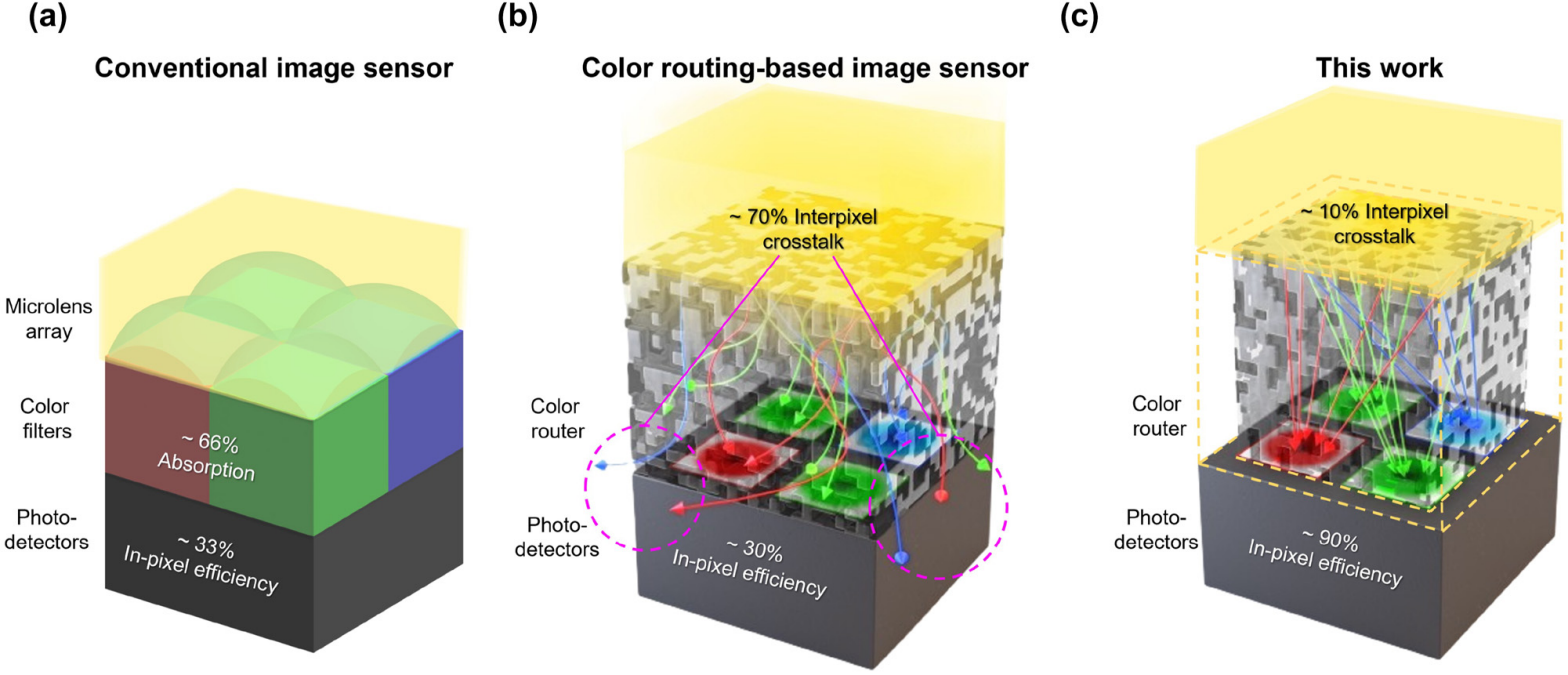
Spectral engineering in CMOS image sensors. (a) Conventional image sensors consist of color filters, a microlens array, and photodetectors, which cap the maximum optical efficiency of image sensors to 33 %. (b) Color routing-based image sensors consisting of a color router and photodetectors demonstrated a peak optical efficiency of 99 %. However, it inherently suffers interpixel optical crosstalk due to the periodic nature of the color routers. (c) The proposed color routing technique highly suppresses interpixel optical crosstalk to less than 10 %.

Complementary metal–oxide–semiconductor (CMOS) image sensors play a pivotal role in a wide range of applications that rely on digital imaging systems [1], such as smartphone cameras [2], [3], automotive cameras [4], [5], and surveillance systems [6], [7]. Typically, a standard CMOS image sensor (CIS) comprises a configuration of microlenses, a color filter array, and photodetectors [8], [9], as shown in Figure 1(a). The microlenses are responsible for gathering incident light, while the color filter selectively permits the transmission of light of wavelengths corresponding to each subpixel while absorbing the others. This process ensures that only the desired wavelengths reach the photodetector.

With ongoing trends toward device miniaturization and the increasing demand for higher resolution, there has been a significant reduction in pixel size, now approaching the submicrometer scale [10], [11]. However, this miniaturization presents several challenges. As pixel size decreases, the volume of light each pixel captures diminishes quadratically. Furthermore, conventional microlensing, which relies on ray optics, becomes less effective at these smaller scales, which fall within the wave optics regime [12], [13]. This inefficiency leads to a reduction in transmission efficiency and quantum efficiency [14] while exacerbating the issue of crosstalk among pixels [15]. Furthermore, a fundamental limitation exists in standard CISs: not all incident light contributes to the electrical signal due to absorption by the color filters. Consequently, in the most optimistic scenario, only 33 % of the incident photons are available for imaging purposes.

The ideal solution for overcoming these losses is to design a device capable of directing incident light to the appropriate photodetector, depending on the photon energies (i.e., colors). These color-routing techniques have demonstrated efficient color separation using various approaches, including single-layer metasurface-based strategies [16], [17], [18], laterally or vertically stacked metasurfaces [19], [20], and inverse design methods [21], [22], [23], [24], [25], [26], [27], [28]. Notably, adjoint optimization-based inverse design techniques [29], [30], [31], [32], [33], [34], [35], [36], [37], [38], [39], [40] achieve remarkable color-routing efficiencies. The 2D color router, for instance, achieves peak efficiencies above 80 % across R, G, B, and IR wavelengths and significantly reduces color crosstalk below 10 % [41]. In 3D configurations, Bayer layout-based color routers exhibit nearly perfect routing efficiencies without loss of photons [42]. However, studies on adjoint optimization-based color routers have demonstrated that they unintentionally direct a significant amount of Poynting vectors in a predominantly horizontal orientation. This lateral orientation increases the likelihood of crosstalk between neighboring pixels, as discussed under “interpixel crosstalk” in this work, potentially degrading image quality in image sensors significantly.

In this paper, we address the pervasive issue of interpixel crosstalk within these color routing-based image sensors. We propose a series of novel structural designs and employ custom incident waves to significantly mitigate this effect (Figure 1(c)). Importantly, our initial findings establish a critical baseline: conventional adjoint optimization-based color routers inherently exhibit significant interpixel crosstalk, with light, originally directed to propagate toward a specific pixel, frequently “leaking” to adjacent pixels, as illustrated in Figure 1(b). This insight underpins the advanced structural designs and methodologies we propose. Our optimized designs achieve a peak in-pixel optical efficiency of 65.1 % and demonstrate a remarkable reduction in interpixel crosstalk (2.6 %). Further enhancement is achieved with the integration of isolation walls, leading to an in-pixel optical efficiency of 87.2 % (interpixel crosstalk of 0.8 %). Such advancements mark our color router as significantly outperforming existing techniques in in-pixel optical efficiency, thereby representing a substantial stride toward the commercialization of next-generation solid-state image sensors.

## 2 Color routers with periodic boundary conditions

This section describes the design of a color router utilizing adjoint optimization and conducts a quantitative assessment of the recurring issue of interpixel crosstalk in the optimized structures, as shown in Figure 1(b). Previous studies have employed the adjoint optimization of color routers under the assumption of Bloch boundary conditions to model the infinite periodic replication of these devices. Although this approach reduces computational overhead, it also increases the vulnerability to interpixel crosstalk due to the periodic replication of the target planes in the color routing.

To rigorously analyze interpixel crosstalks of the pioneer studies, we reproduce the most extensively examined 2D color router design as shown in Figure 3. Figure 3(c) shows a designable region, 2 μm height and 1.5 μm periodicity, composed of SiO_2_ and Si_3_N_4_. With a minimum feature size of 10 nm, the designable region can be represented as 200 layers of SiO_2_ and Si_3_N_4_. The designable region is directly connected to the photodetectors, where 0.2 μm width Deep Trench Isolation (DTI) separates them. The width of the photodetectors is assumed to be 0.3 μm, smaller than those employed in the conventional image sensors [43], [44], [45]. For reproducing purposes, dielectric constants are assumed 2.1 for SiO_2_, 4.0 for Si_3_N_4_, and 5.0 for the photodetector across the visible spectrum. The finite-difference time-domain simulations are conducted using the open-source solver, Meep [46], with a grid spacing set at 10 nm, resulting in 30,000 design parameters in a 2 μm × 1.5 μm design region.

**Figure 2:**
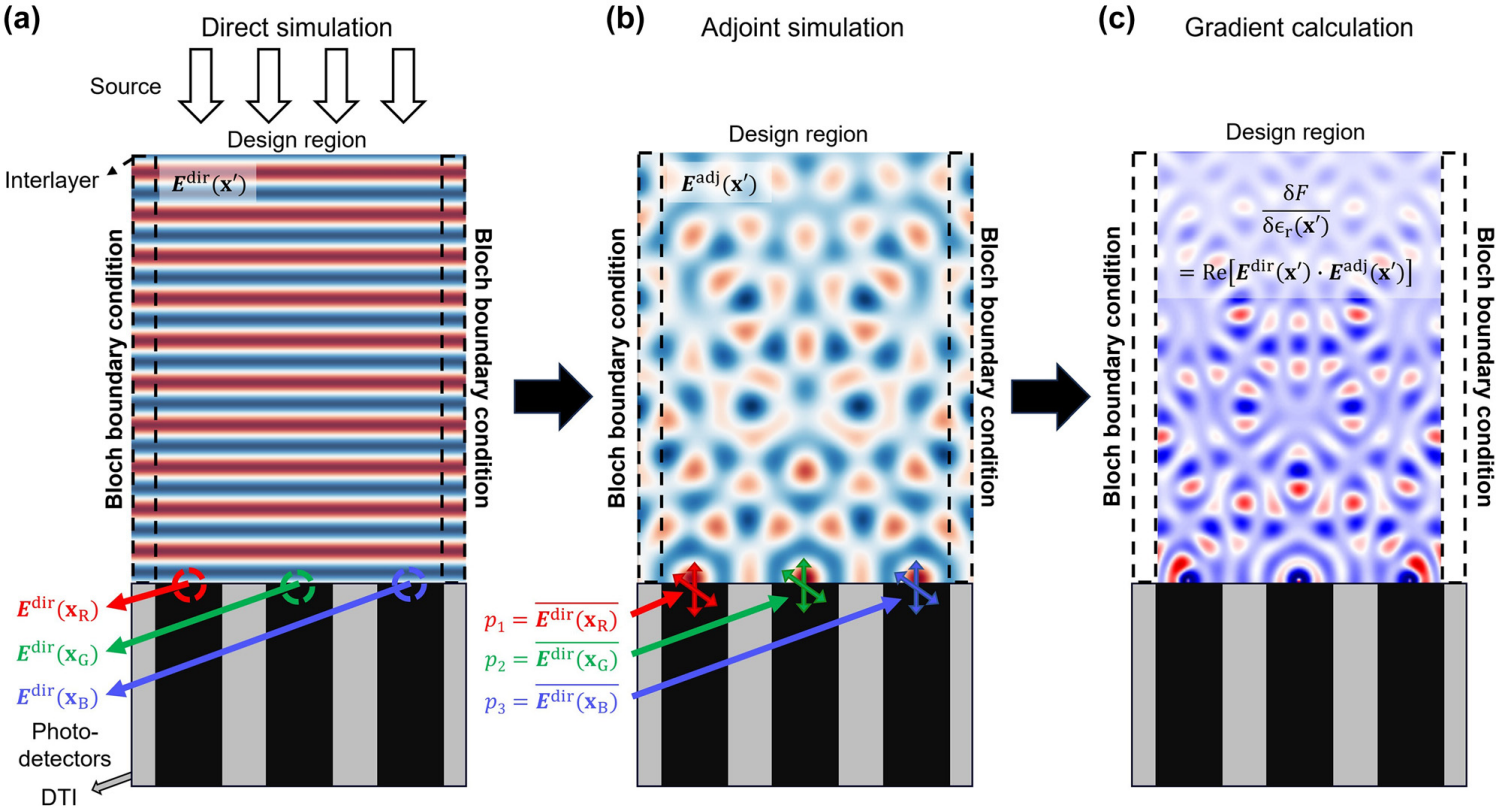
Schematic of the adjoint optimization for designing CMOS image sensors. (a) Schematic depiction of direct simulation of adjoint optimization. A plane wave (or Gaussian beam) is excited from the free space while ***E*** ^dir^(**x**_R_), ***E*** ^dir^(**x**_G_), and ***E*** ^dir^(**x**_B_) are saved for the adjoint simulation at the center of the photodetectors. In addition, electric fields at the designable region (***E*** ^dir^(**x**′)) are stored for gradient calculation. (b) Dipoles with amplitudes of $\bar{E^{\text{dir}}\left(x_{R}\right)}$ , $\bar{E^{\text{dir}}\left(x_{G}\right)}$ , and $\bar{E^{\text{dir}}\left(x_{B}\right)}$ are excited and back-propagated through the designable region to obtain ***E*** ^adj^(**x**′) (adjoint fields). (c) Gradients with respect to the geometric degree of freedom can be calculated only with two simulations (direct and adjoint simulations).

In this study, we define the figure of merit (FOM) of the adjoint optimization as an electric field intensity at the center of each subpixel, as shown in Figure 2. It might lead to a mismatch between optical efficiency and the FOM. However, the suggested FOM offers the advantage of centering the fields, thus mitigating the risk of electrical crosstalk while concurrently simplifying the adjoint simulation process. Therefore, our figure of merit is formulated as:

(1) $$\begin{array}{ll} & F=\alpha \int {\left|E\left(x_{R},\lambda \right)\right|}^{2}d\lambda +\beta \int {\left|E\left(x_{G},\lambda \right)\right|}^{2}d\lambda \\ & \;+\gamma \int {\left|E\left(x_{B},\lambda \right)\right|}^{2}d\lambda \end{array}$$

where **x**_R_, **x**_G_, **x**_B_ denote the centers of photodetectors in each subpixel, and ***E*** represents the electric field. We segment visible wavelengths into three regimes: 400–500 nm for red (R), 500–600 nm for green (G), and 600–700 nm for blue (B), respectively. The field intensity is aggregated at evenly spaced wavelengths within these bands to derive an average efficiency for each spectral range. The coefficients *α*, *β*, and *γ* serve as normalization factors for each color.

Figure 2 shows the schematic of the adjoint optimization process used in this study. Initially, the FOM value for each wavelength is calculated through direct simulation, where the incident plane wave is excited at the top of the design region as shown in Figure 2(a). In the direct simulation, the electric fields at the center of photodetectors (***E*** ^dir^(**x**_R_), ***E*** ^dir^(**x**_G_), and ***E*** ^dir^(**x**_B_)) are computed and utilized as amplitudes of the adjoint sources, while $E^{\text{dir}}\left(x^{\prime }\right)$ at the design area is saved for the gradient calculation. Subsequently, the adjoint sources are computed through $J_{\text{adj}}=-iwP_{\text{adj}}=-iw\partial F/\partial E$ , the amplitude of the adjoint dipole source thereby being $\bar{E^{\text{dir}}\left(x_{R}\right)},\bar{E^{\text{dir}}\left(x_{G}\right)},\bar{E^{\text{dir}}\left(x_{B}\right)}$ , respectively. The adjoint dipole sources back-propagate through the designable region and generate $E^{\text{adj}}\left(x^{\prime }\right)$ as shown in Figure 2(b). The adjoint dipole sources are evenly distributed across wavelengths ranging from 400 to 700 nm, accompanied by normalization factors *α*, *β*, and *γ* as outlined in (1). Finally, the gradient of the FOM with respect to changes in the permittivity parameter *ϵ*_*r*_(**x**′) within the design region is computed as *δF*/(*δϵ*_*r*_(**x**′)) = Re[***E*** ^dir^(**x**′) ***E*** ^adj^(**x**′)] (Figure 2(c)). This iterative process continues until the improvement in the performance metric converges, resulting in an efficient color routing structure.

In previous studies using inverse design methods for designing color routers, optical efficiency calculations included the light absorbed by the photodetectors located in neighboring subpixels beyond periodic boundaries. This resulted in interpixel crosstalk, as light directed toward adjacent pixels could augment efficiency gains if absorbed by photodetectors with the corresponding wavelength. In this study, we quantify interpixel crosstalk by calculating the proportion of light absorbed by adjacent pixels relative to the incident power. Two materials, specifically SiO_2_ and Si_3_N_4_, which are well suited for standard CMOS fabrication [47], [48], were chosen to realize color routing within multilayer structures. Expressing SiO_2_ and Si_3_N_4_ as material densities (*ρ*), where *ρ* = 0 denotes SiO_2_ and *ρ* = 1 indicates Si_3_N_4_, the initial configuration of the design region was initialized with a random material density distribution between 0.45 and 0.55. A linear convolution filter is applied to convert the material densities (*ρ*) to binary values [49]:

$$\rho^{\prime }=\omega \left(x\right)*\rho$$

where *ρ*′ represents the filtered material density, *ω* denotes the conic filter kernel [50], and ∗ is a multidimensional convolution. In order to apply minimum feature size constraint, the conic filter kernel is applied. Subsequently, the filtered material density is projected to approximate binary values using a hyperbolic tangent function.

$$\rho^{\prime \prime }=\frac{\tanh\left(\beta \eta \right)+\tanh\left(\beta \left(\rho^{\prime }-\eta \right)\right)}{\tanh\left(\beta \eta \right)+\tanh\left(\beta \left(1-\eta \right)\right)}$$

*β* is thresholding parameter that determines the degree of binarization, *η* is a threshold point, which has a value of 0.5, and *ρ*″ is the projected material density [51].

**Figure 3:**
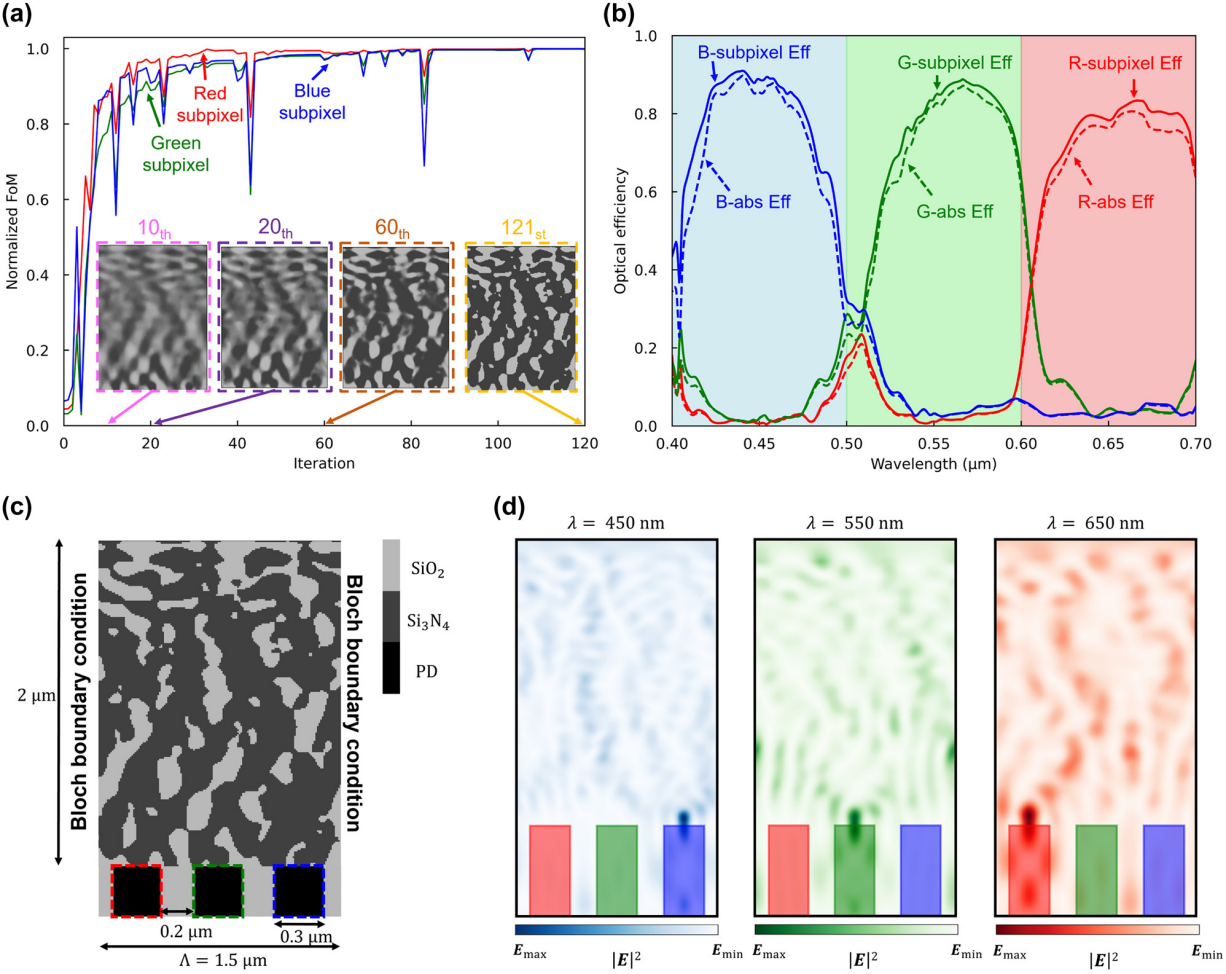
Adjoint optimization of the 2D color router with periodic boundary condition. (a) A normalized figure of merit for red, green, and blue subpixels versus the adjoint optimization iterations. The design region is represented as a grayscale permittivity ranging from SiO_2_ and Si_3_N_4_ permittivities. The inset figures (pink, purple, and brown dashed boxes) indicate intermediate design parameters and the yellow-dashed inset figure represents the optimized design parameters. (b) The optical efficiency of the three subpixels at the visible wavelengths, including interpixel crosstalk. The curves indicate transmission normalized optical efficiency, while dashed curves indicate absolute optical efficiency. (c) The 2D optimized color routing-based CMOS image sensor. It has 0.3 μm width of the photodetectors and 0.2 μm width of the deep trench isolation. The design region has 1.5 μm periodicity and 2 μm height, which gives 30,000 number of design parameters when the grid spacing is 10 nm. (d) Intensity profiles of the optimized image sensor at three representative wavelengths (450, 550, 650 nm).

As the design undergoes adjoint optimization and the material density is adjusted through gradient descent, the FOM notably enhances, as illustrated in Figure 3(a). We utilize the Method of Moving Asymptotes (MMA) algorithm, which is based on the conservative convex separable approximation algorithm [52], to update the material density function using gradient information. This algorithm incorporates a nonlinear constant to avoid trapping into the local minima, but it sometimes causes an abrupt drop in FOM. The figure portrays the intensity of light incident upon the central areas of the R, G, and B subpixels. Figure 3(c) shows the optimized 2D color router structure, which exclusively comprises SiO_2_ and Si_3_N_4_ with material densities binarized to 0 and 1. The optimized color router design achieves outstanding optical efficiency. However, it leaves the question of how many photons may be directed to the designated pixel (not the adjacent pixel) due to the horizontally connected Si_3_N_4_ structures.

We evaluate the performance of the optimized color router by calculating the transmission normalized optical efficiency (*OE*(*i*) = *T*(*x*_*i*_, *λ*)/(*x*_*T*_, *λ*)) for each color subpixel (*i* = R, G, B), which is the ratio of the light transmitted to the appropriate subpixel (*T*(*x*_*i*_, *λ*)) out of the total light transmitted to the entire pixel region (*T*(*x*_*T*_, *λ*), *x*_*T*_ = *x*_Total_). The results, presented in Figure 3(b), demonstrate a transmission normalized optical efficiency exceeding 80 %. Additionally, the absolute optical efficiency (*OE*(*i*) = *T*(*x*_*i*_, *λ*)/*P*(*λ*)), representing the ratio of light transmitted to the appropriate subpixel *T*(*x*_*i*_, *λ*) relative to the incident light on the entire device (*P*(*λ*)), was maintained at a remarkably high level. We demonstrate two different definitions of the optical efficiency to address various metrics of the image sensors. Depending on the applications, each definition can be used to evaluate the sensor’s performance. Figure 3(d) depicts the field intensity profile for three representative wavelengths (*λ* = 450, 550, and 650 nm) corresponding to R, G, and B colors. It demonstrates the effective focusing of light onto the target subpixels at each wavelength.

**Figure 4:**
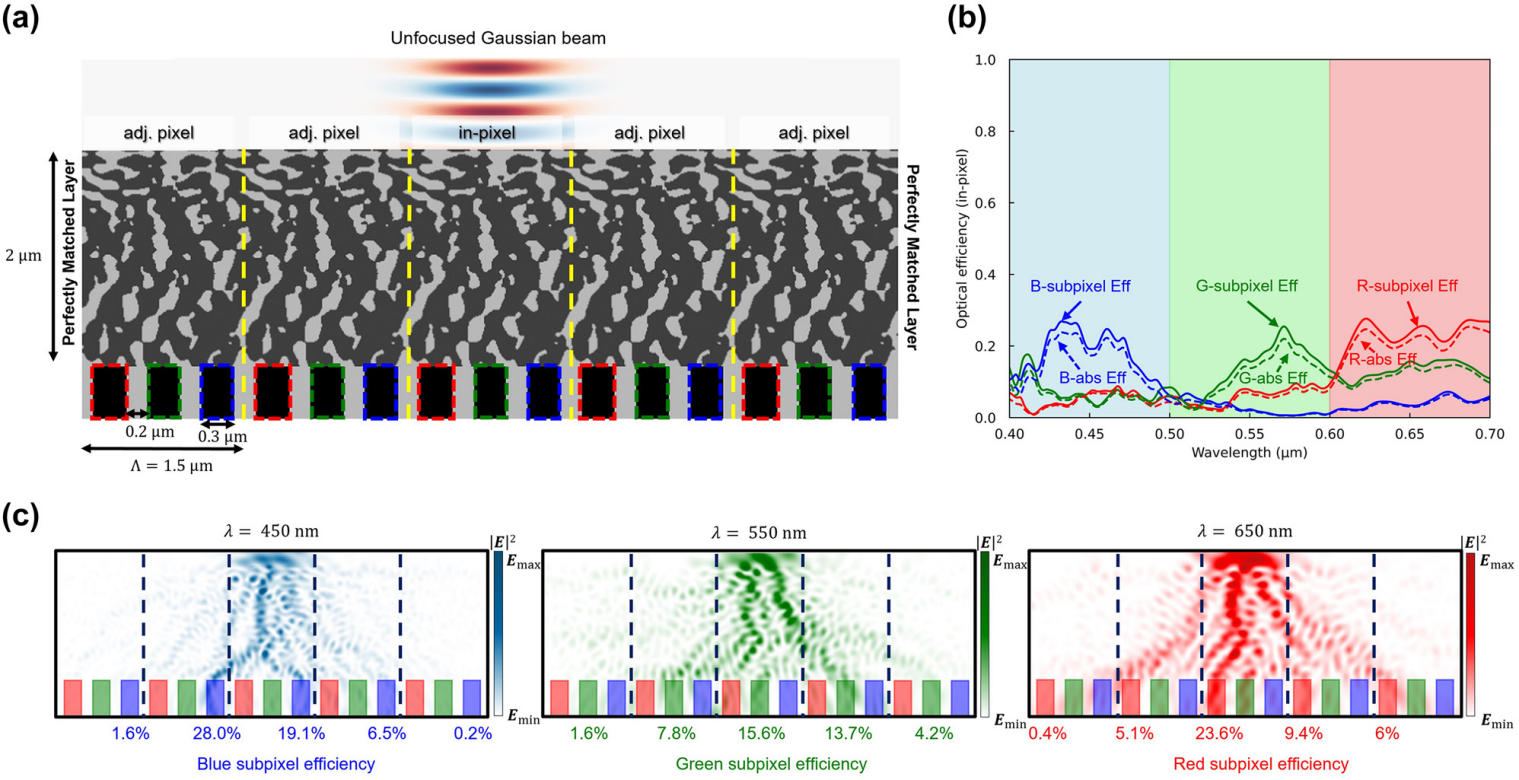
Interpixel crosstalk calculation based on unfocused Gaussian beam. (a) The optimized periodic structure is cut into five periods and simulated with PML boundary conditions. The unfocused Gaussian beam is used to calculate an approximated interpixel crosstalk where the beam mimics normal incident plane wave into the single pixel. (b) The optical efficiency of the three subpixels (at the center pixel) over the visible wavelengths. The curves indicate transmission normalized optical efficiency, while dashed curves indicate absolute optical efficiency. The overall optical efficiencies have dropped significantly due to the interpixel crosstalk. (c) Intensity profiles of the five periods of the optimized image sensor at three representative wavelengths (450, 550, and 650 nm). More than half of the incident power is delivered to the adjacent subpixels, indicating significant interpixel crosstalk.

To assess the interpixel crosstalk of the designed color router, we introduced an unfocused Gaussian beam into the central unit cell of a supercell, which consists of the designed multilayer color router replicated over five units, as depicted in Figure 4(a). The unfocused Gaussian beam, designed with a beam waist of 0.75 μm – approximately half the dimension of the unit cell – was configured to generate a planar wavefront, effectively simulating a direct plane wave incident on the central unit cell. This setup emulates the incidence of a plane wave specifically targeting the central unit cell. The definition of the optical efficiency in color routers is not unique. For example, a single layer-based color router may calculate its optical efficiency separately for each color subpixel [19], [53]. However, we define our optical efficiency based on the unit cell region for simplicity and a fair comparison against existing works. Figure 4(b) illustrates the percentage of the broadband unfocused Gaussian beam absorbed into the target subpixel of the central unit cell, defined as in-pixel optical efficiency. The peak in-pixel optical efficiency of red, green, and blue subpixels was 27.6 %, 25.5 %, and 26.9 %, respectively, and the average efficiency was 23.2 %, 13.1 %, and 18.1 %.

These findings show a marked discrepancy from the optical efficiencies illustrated in Figure 3(b), suggesting that interpixel crosstalk may erroneously appear to enhance optical efficiency in those measurements. This divergence highlights the vulnerability of inversely designed color routers to pronounced interpixel crosstalk. Figure 4(c) further emphasizes this, indicating that only 23.6 %, 15.6 %, and 19.1 % of light entered the photodetectors of the red, green, and blue subpixels in the central unit cell, respectively, with a larger proportion infiltrating the subpixels of neighboring unit cells. Notably, at a wavelength of 450 nm, the quantity of light directed to an adjacent unit cell (28.0 %) surpasses that directed to the in-pixel (19.1 %).

Our validation confirms that conventional color routers designed by combining the Bloch boundary and inverse design may suffer from significant interpixel crosstalk. This issue significantly undermines the signal-to-noise ratio (SNR) of CMOS image sensors and complicates image processing operations. The interpixel crosstalk induces a discrepancy between the expected and observed levels of light absorption by photodetectors, resulting in image blurring. Furthermore, integrating compensation algorithms into image processing presents a significant challenge, as the extent of interpixel crosstalk fluctuates with both wavelength and the spatial arrangement of pixels. This phenomenon contributes to the degradation in imaging resolution, which is a pivotal aspect in systems that depend on precise spatial information. Addressing the issue of interpixel crosstalk is essential for enabling color routers to replace traditional components such as color filters and microlens arrays in CMOS image sensor applications.

## 3 Interpixel crosstalk-minimized designs

In this section, we propose two strategies to mitigate the interpixel crosstalk within color routers optimized via inverse design methods under Bloch boundary conditions. The initial approach involves the insertion of a physical interlayer between the design region and the boundary condition to minimize interpixel crosstalk. The materials employed for the interlayer include tungsten or air, which have been utilized in CMOS image sensor processes for color filter isolation [54], [55]. This method substantially reduces interpixel crosstalk and enhances imaging resolution, albeit at the expense of elevated manufacturing costs due to the interlayer incorporation. Conversely, the alternative method employs PML boundary conditions in the horizontal orientation of the design region. Here, any light that spills into neighboring pixels is treated as lost, allowing for a configuration that minimizes interpixel crosstalk. This approach offers notable benefits in reducing manufacturing complexity and costs as it obviates the need for an interlayer.

### 3.1 Color routers with physical interlayers

**Figure 5:**
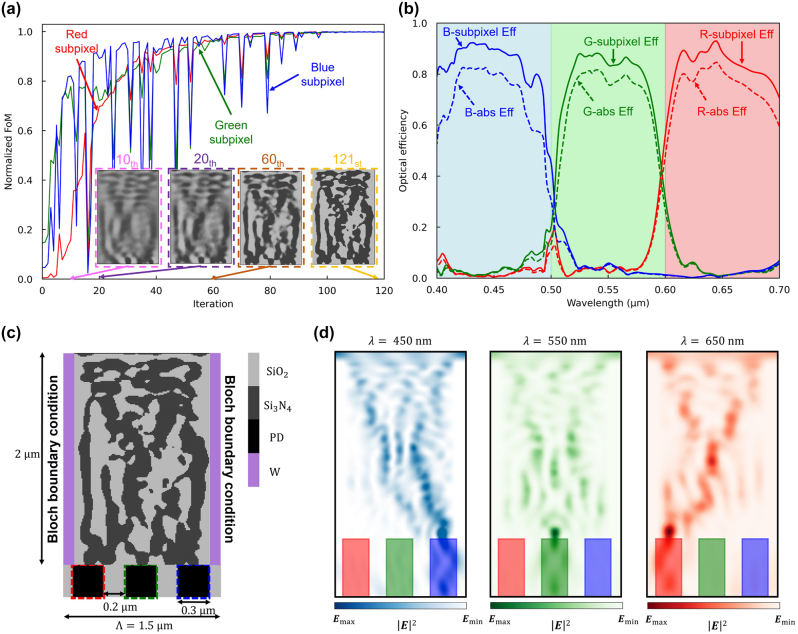
Adjoint optimization of the 2D color router with tungsten isolation walls. (a) A normalized figure of merit for red, green, and blue subpixels versus the adjoint optimization iterations. The design region is represented as a grayscale permittivity with values between SiO_2_ and Si_3_N_4_. The inset figures (pink, purple, and brown dashed boxes) indicate intermediate design parameters and the yellow-dashed inset figure represents the optimized design parameters. (b) Optical efficiency of the three subpixels over the visible wavelengths. The curves indicate transmission normalized optical efficiency, while dashed curves indicate absolute optical efficiency. The high absorption rate of tungsten causes the difference between the normalized and absolute efficiency. (c) The 2D optimized color routing-based CMOS image sensor with the proposed color routing technique. It has 0.3 μm width of the photodetectors and 0.2 μm width of the deep trench isolation. The design region, which includes 0.1 μm tungsten gaps on both sides, has 1.5 μm periodicity and 2 μm height, giving 26,000 number of design parameters when the grid spacing is 10 nm. (d) Intensity profiles of the optimized image sensor at three representative wavelengths (450, 550, and 650 nm).

As a primary strategy to mitigate interpixel crosstalk, we performed adjoint optimization with tungsten isolation walls laterally along the design region. Tungsten was selected for its compatibility with existing CMOS fabrication technology and its ease of integration [56], [57]. The dielectric constant of tungsten used in the simulation was obtained from the existing source [58] and fitted with Drude-Lorentz model [59]. As shown in Figure 5(c), Bloch boundary conditions were applied, positioning the tungsten isolation walls at a height of 2 μm – aligned with the height of the design area – and a width of 0.1 μm on each flank of the design region, corresponding to the width of the DTI [60]. With a 0.2 μm interlayer spacing between successive design regions, the width of the design area was reduced to 1.3 μm, while maintaining a periodicity of 1.5 μm. The design region was encapsulated by tungsten isolation wall, and through adjoint optimization, a color router composed of SiO_2_ and Si_3_N_4_ was designed. Figure 5(a) illustrates the variation of the FOM for R, G, and B wavelengths over iterations. Initialized with a grayscale material density, the design region progressively evolved toward a binary structure of Si_3_N_4_ and SiO_2_ with increasing iterations. The optimized color router demonstrates the successful routing of incident light to the R, G, and B subpixels, demonstrating average optical efficiencies of 83.5 %, 75.2 %, and 84.4 % and peak optical efficiencies of 93.0 %, 88.2 %, and 92.3 %, respectively, as shown in Figure 5(b). The structural design of the optimized color router, as shown in Figure 5(c), as configured with disconnected elements of tungsten and Si_3_N_4_, diverging from the traditional horizontally connected designs depicted in Figure 3(c). This design is anticipated to substantially reduce interpixel crosstalk while concurrently minimizing the absorption loss by tungsten. Figure 5(d) illustrates the field intensity profile when a plane wave is incident on the optimally designed color router at center wavelengths of 450, 550, and 650 nm. A significant portion of the incident light is directed toward the desired subpixel, indicating a potential reduction in light leakage to neighboring pixels. We propose an efficient method to mitigate interpixel crosstalk without considerably compromising the efficiency of color routing through the incorporation of the tungsten isolation wall.

**Figure 6:**
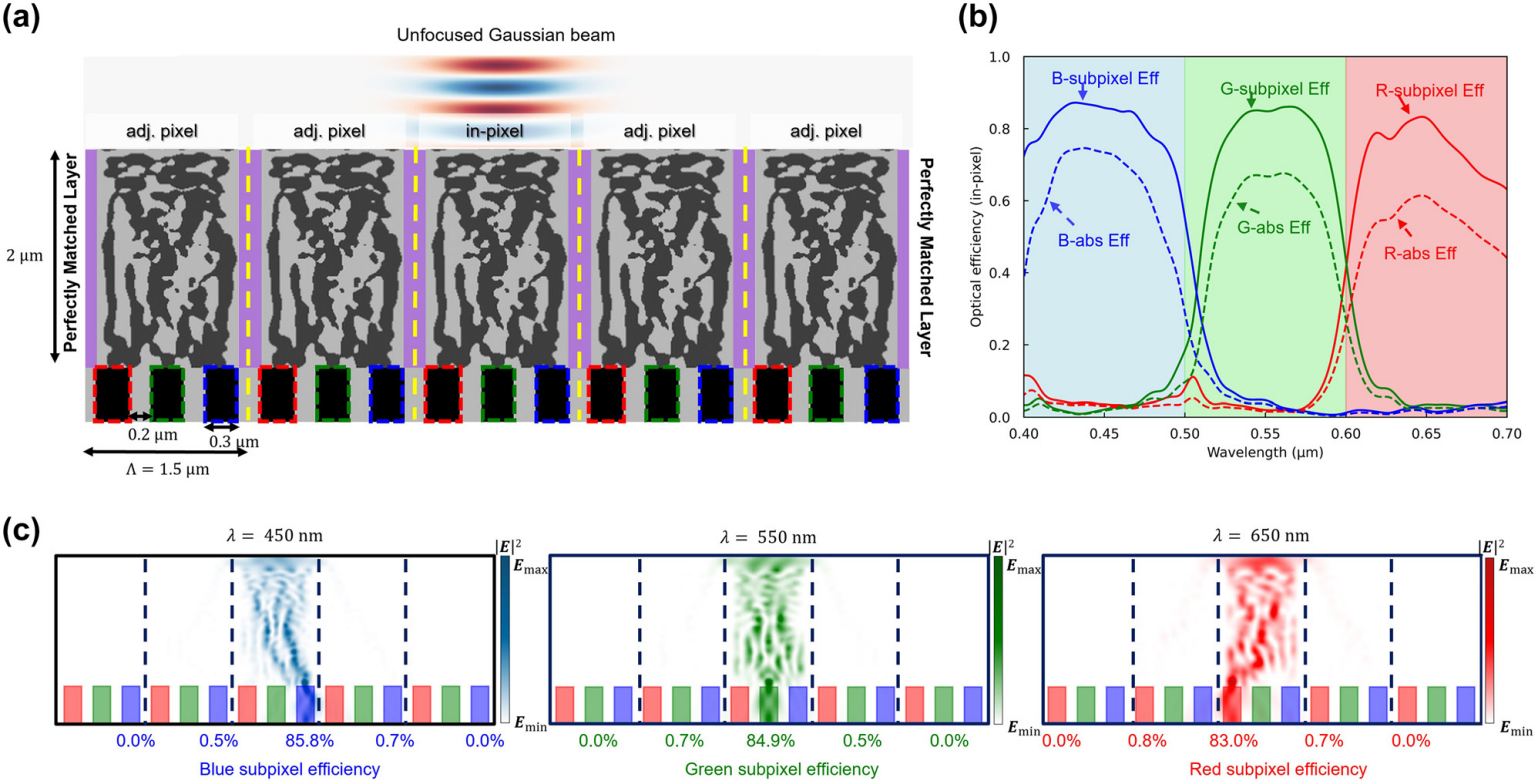
Interpixel crosstalk calculation based on unfocused Gaussian beam. (a) The optimized periodic structure is cut into five periods and simulated with PML boundary conditions. The unfocused Gaussian beam is used to calculate an approximated interpixel crosstalk where the beam mimics normal incident plane wave into the single pixel. (b) The optical efficiency of the three subpixels (at the center pixel) over the visible wavelengths. The curves indicate transmission normalized optical efficiency, while dashed curves indicate absolute optical efficiency. The proposed color routing technique significantly reduced the optical efficiency loss due to interpixel crosstalk, while the high absorption of tungsten contributes to the difference between absolute and normalized efficiency. (c) Intensity profiles of the five periods of the optimized image sensor at three representative wavelengths (450, 550, 650 nm). The incident power is effectively prevented from entering the adjacent subpixels, indicating successful suppression of interpixel crosstalk.

The quantification of interpixel crosstalk was conducted using Gaussian beam and super-cell configurations. The optimized color routers were replicated into five units, and an unfocused Gaussian beam, identical to that used in Figure 4(a), was directed onto the central unit cell, as shown in Figure 6(a). Figure 6(b) illustrates the in-pixel optical efficiency, which represents the light absorbed by the photodetector positioned within the central unit cell upon incidence of the Gaussian beam. The calculated average optical efficiencies for the red, green, and blue subpixels were 73.6 %, 71.3 %, and 81.1 %, respectively, with corresponding peak efficiencies of 83.3 %, 86.1 %, and 87.2 %, respectively. The inclusion of the tungsten interlayer led to an increase in in-pixel optical efficiency by 50.4 %, 58.2 %, and 63.0 %, respectively, compared to the color router without an interlayer, signifying effective suppression of interpixel crosstalk. Figure 6(c) illustrates the field intensity profiles at the central wavelengths for each color, revealing notably high color routing efficiencies of 83.0 %, 84.9 %, and 85.8 %, respectively, at the central unit cell. In comparison, the proportion of light entering the subpixels of adjacent unit cells remained below 1.5 %. Nevertheless, Figure 6(b) indicates a decrease in absolute optical efficiency of up to 20 % compared to the transmission normalized optical efficiency. This decrease is attributed to absorption and reflection losses induced by tungsten components.

**Figure 7:**
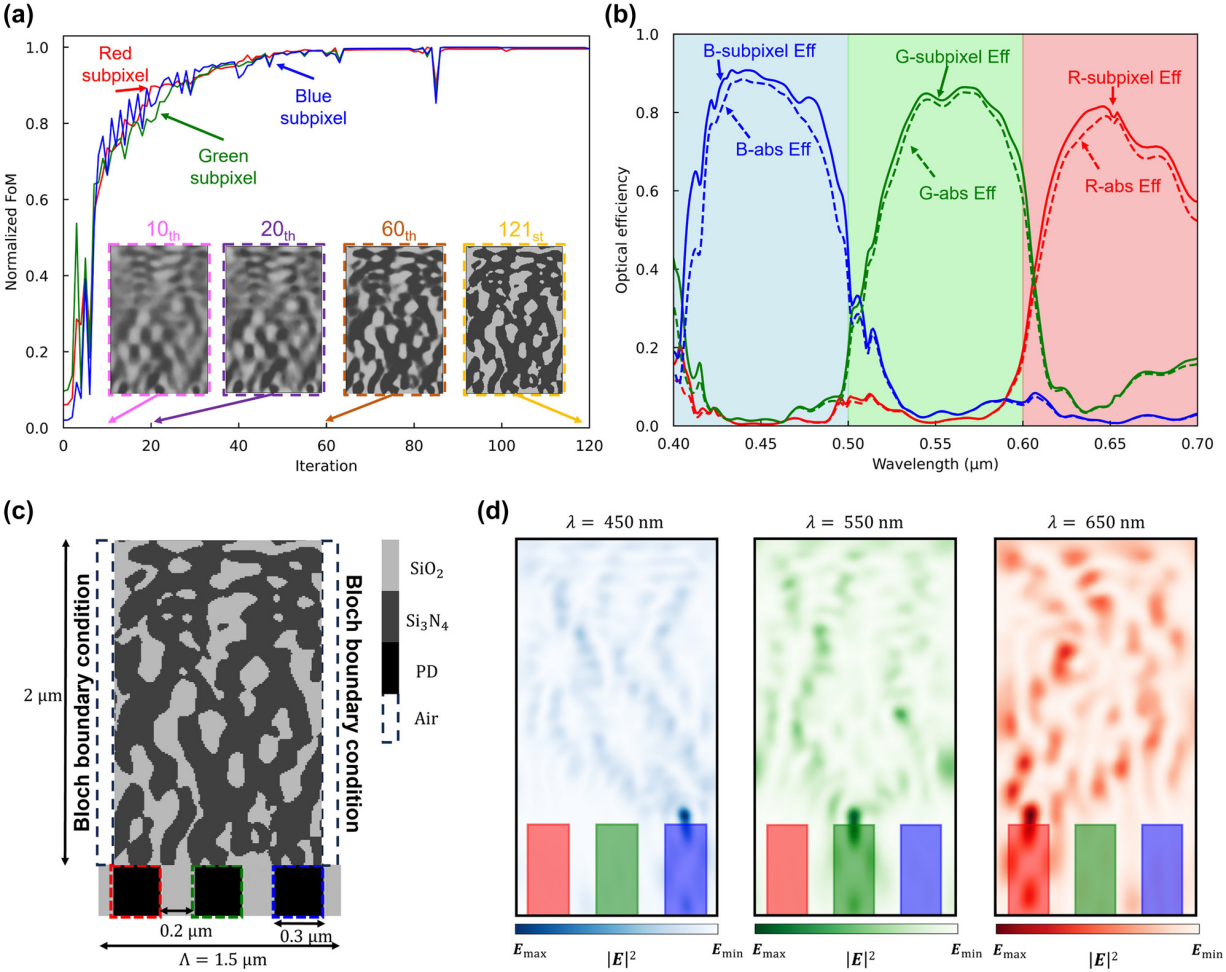
Adjoint optimization of the 2D color router with air gaps. (a) A normalized figure of merit for red, green, and blue subpixels versus the adjoint optimization iterations. The design region is represented as a grayscale permittivity with values between SiO_2_ and Si_3_N_4_. The inset figures (pink, purple, and brown dashed boxes) indicate intermediate design parameters and the yellow-dashed inset figure represents the optimized design parameters. (b) Optical efficiency of the three subpixels over the visible wavelengths. The curves indicate transmission normalized optical efficiency, while dashed curves indicate absolute optical efficiency. (c) The 2D optimized color routing-based CMOS image sensor with the proposed color routing technique. It has 0.3 μm width of the photodetectors and 0.2 μm width of the deep trench isolation. The design region, which includes 0.1 μm air gaps on both sides, has 1.5 μm periodicity and 2 μm height, giving 26,000 number of design parameters when the grid spacing is 10 nm. (d) Intensity profiles of the optimized image sensor at three representative wavelengths (450, 550, 650 nm).

While the use of tungsten isolation walls effectively suppresses interpixel crosstalk, the inherent optical losses attributable to tungsten’s high absorption rate pose a significant challenge. To counteract the absorption losses associated with tungsten, we propose a novel design approach for the color router, which aims to mitigate interpixel crosstalk while concurrently minimizing optical loss by integrating an air gap instead of tungsten [54]. The structural configuration of the color router remains consistent with the previously designed tungsten-based interlayer color router, with the primary modification being the substitution of tungsten with an air gap. Figure 7(a) illustrates that the design region undergoes optimization utilizing the adjoint optimization technique, resulting in the optimized color router composed of SiO_2_ and Si_3_N_4_, as depicted in the inset figure (yellow dashed box). Figure 7(b) indicates that the optimized color router shows a high transmission-normalized optical efficiency, exceeding 80 % for the B and G subpixels and surpassing 70 % for the R subpixel. Substituting tungsten with air reduces the disparity between transmission-normalized and absolute optical efficiency, attributed to the reduced absorption and reflection losses inherent to tungsten. The optimized color router, shown in Figure 7(c), maintains structural similarity to the design with the tungsten interlayer, except for some direct contact between Si_3_N_4_ and the air gap. Figure 7(d) presents the field intensity profile upon incidence of a plane wave on the optimized color router for three representative wavelengths (450, 550, 650 nm), demonstrating efficient focusing of field intensity to the respective subpixels without color crosstalk.

**Figure 8:**
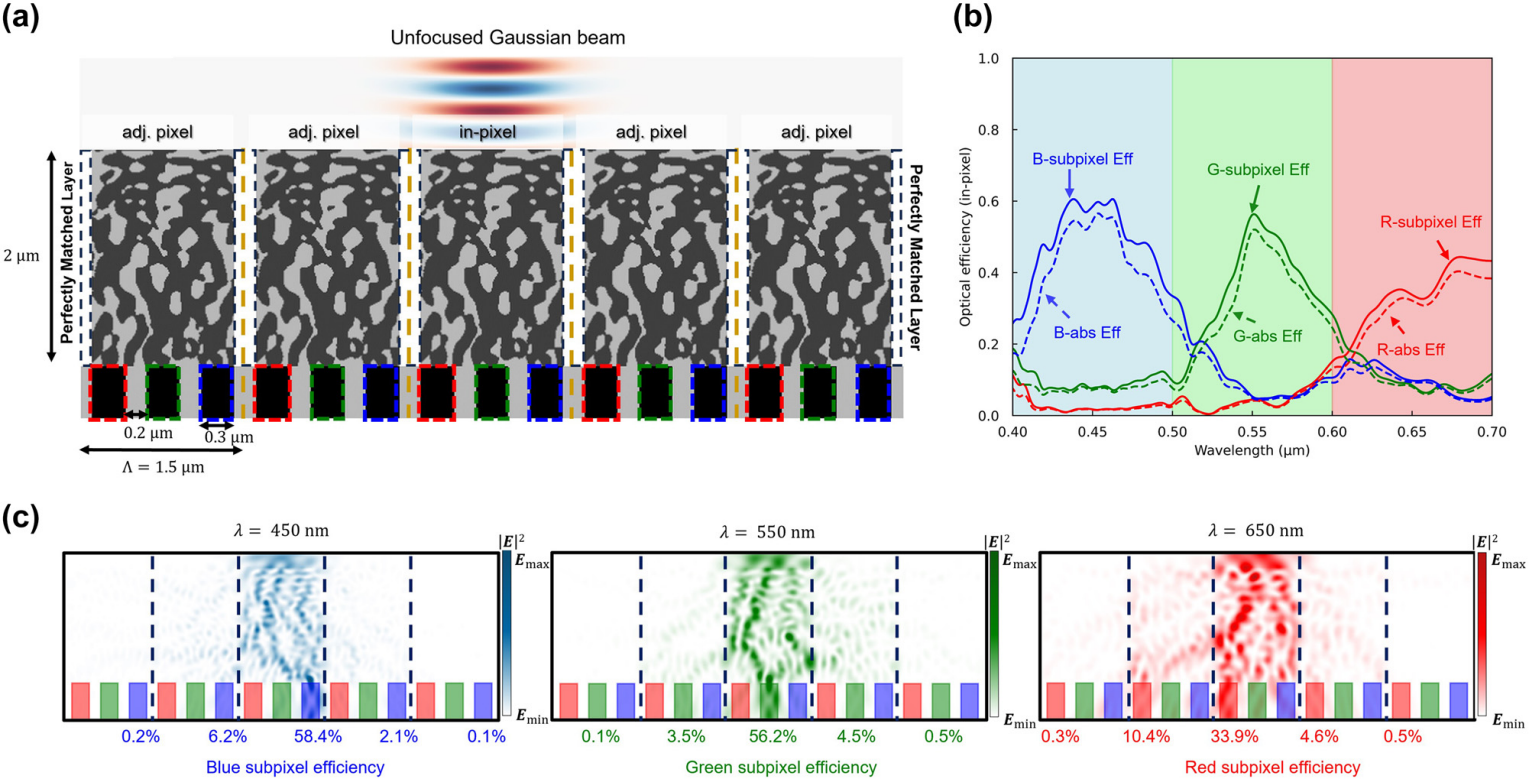
Interpixel crosstalk calculation based on unfocused Gaussian beam. (a) The optimized periodic structure is cut into five periods and simulated with PML boundary conditions. The unfocused Gaussian beam is used to calculate an approximated interpixel crosstalk where the beam mimics normal incident plane wave into the single pixel. (b) The optical efficiency of the three subpixels (at the center pixel) over the visible wavelengths. The curves indicate transmission normalized optical efficiency, while dashed curves indicate absolute optical efficiency. The proposed color routing technique significantly reduced the optical efficiency loss due to interpixel crosstalk compared to Figure 4(b). (c) Intensity profiles of the five periods of the optimized image sensor at three representative wavelengths (450, 550, 650 nm). The incident power is effectively prevented from entering the adjacent subpixels, indicating successful suppression of interpixel crosstalk.

We observed that the color router featuring the air gap exhibits notable optical efficiency. To further verify the occurrence of interpixel crosstalk, we employed the Gaussian beam and super-cell configurations used in the previous simulations, as shown in Figure 8(a). Figure 8(b) illustrates the in-pixel optical efficiency, representing the quantity of light absorbed by the photodetector placed within the unit cell when a Gaussian beam is incident on the central unit cell. The average optical efficiencies computed for the red, green, and blue subpixels were 33.1 %, 34.6 %, and 48.0 %, respectively, with corresponding peak efficiencies of 44.4 %, 56.4 %, and 60.6 %. This result demonstrates an enhancement in in-pixel optical efficiency by 9.9 %, 21.5 %, and 29.9 % compared to the color router lacking an interlayer, albeit with lower optical efficiency than the color router based on the tungsten interlayer. Figure 8(c) depicts the field intensity profile at the central wavelength for each color, revealing color routing efficiencies of 33.9 %, 56.2 %, and 58.4 % within the central unit cell, with the percentage entering the subpixels of adjacent unit cells all suppressed below 10 %. This investigation suggests that utilizing tungsten and air gap as interlayers can effectively mitigate interpixel crosstalk. In addition to these two materials, implementing materials with high dielectric constants in low-cost processes is expected to serve as promising alternatives for reducing interpixel crosstalk.

### 3.2 Color routers with Gaussian beam

Having demonstrated the effectiveness of tungsten and air gaps as interlayers in mitigating interpixel crosstalk, it becomes pertinent to also explore alternative methodologies that could further streamline the fabrication process by eliminating the need for additional layers. Consequently, we propose a novel approach that utilizes a customized incident source and specific boundary conditions to minimize interpixel crosstalk without the dependency on complex interlayer structures. This strategy aims not only to maintain high optical efficiency but also to simplify manufacturing and reduce production costs.

**Figure 9:**
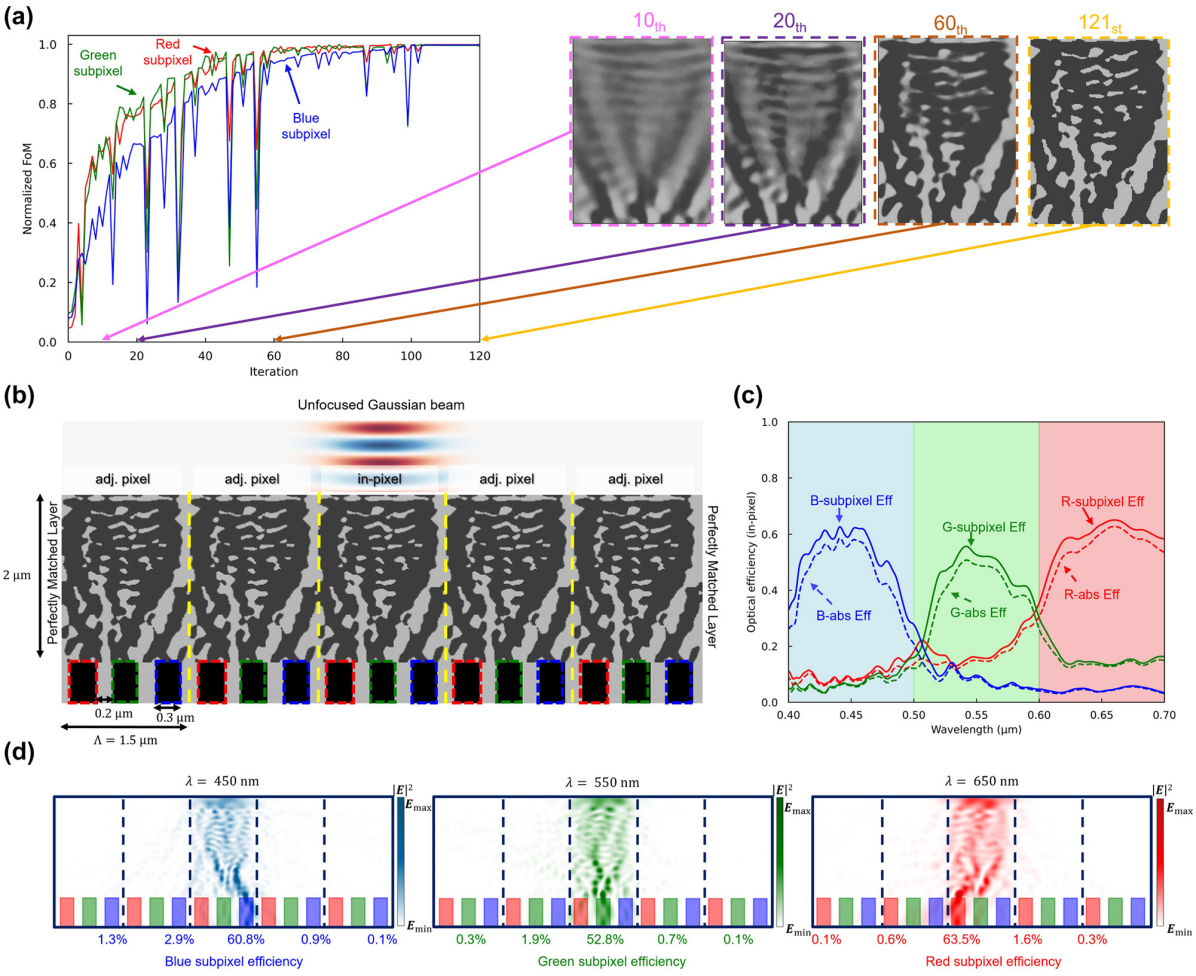
Adjoint optimization of the 2D color router with unfocused Gaussian beam. (a) A normalized figure of merit for red, green, and blue subpixels versus the adjoint optimization iterations. The design region is represented as a grayscale permittivity with values between SiO_2_ and Si_3_N_4_. The inset figures (pink, purple, and brown dashed boxes) indicate intermediate design parameters and the yellow-dashed inset figure represents the optimized design parameters. (b) The optimized periodic structure is cut into five periods and simulated with PML boundary conditions. The unfocused Gaussian beam is used to calculate an approximated interpixel crosstalk where the beam mimics normal incident plane wave into the single pixel. (c) The optical efficiency of the three subpixels (at the center pixel) over the visible wavelengths. The curves indicate transmission normalized optical efficiency, while dashed curves indicate absolute optical efficiency. The structure optimized using an unfocused Gaussian beam demonstrates high efficiency even without an interlayer. (d) Intensity profiles of the five periods of the optimized image sensor at three representative wavelengths (450, 550, 650 nm). The incident power is effectively prevented from entering the adjacent subpixels, indicating successful suppression of interpixel crosstalk.

Prior investigations have employed plane waves as incident waves within a Bloch boundary condition framework for color router optimization. In such configurations, minimizing interpixel crosstalk proved to be challenging, as incident plane waves positively contributed to the optical efficiency even when absorbed by the photodetectors of neighboring pixels. To address this challenge, we propose the placement of PMLs on both sides of a structure consisting of a repeated design area measuring 1.5 μm in width and 2 μm in height. This structure can then be optimized using a customized incident source, as depicted in Figure 9(b). Our customized incident wave utilizes an unfocused Gaussian beam with a diameter matching the width of the unit cell (1.5 μm). This beam configuration, which emulates a plane wave incident on a centrally positioned unit cell, minimizes the influence of incident waves on neighboring unit cells and their penetration into the central unit cell. We conducted an adjoint optimization employing this source and boundary conditions, as outlined in Figure 9(a). In this adjoint optimization, the FOM is defined solely at the central pixel for the purpose of minimizing interpixel crosstalk. However, the iterated design area, including the central pixel and adjacent pixels, undergoes equal adjustments with the geometric modifications of the central pixel in each iteration. Following 121 iterations, the optimization yielded a color router comprising SiO_2_ and Si_3_N_4_, as shown in Figure 9(a). As depicted in Figure 9(b), the optimized structure exhibits minimal horizontally connected regions compared to Figure 4(a), where designs employ plane waves and Bloch boundary conditions. Notably, at the boundaries between unit cells, SiO_2_ is observed to form with a specific thickness, seemingly acting as an interlayer. To quantify the interpixel crosstalk of the optimized color router, we evaluated its in-pixel optical efficiency. On average, the optimized color router demonstrated an in-pixel optical efficiency exceeding 60 %, effectively suppressing interpixel crosstalk without necessitating additional interlayer processes, as depicted in Figure 9(c). Comparative analysis with color routers designed under plane wave and Bloch boundary conditions revealed an increase in in-pixel optical efficiency by 28.7 %, 30.0 %, and 40.5 %, respectively. Figure 9(d) presents the field intensity profile for three representative wavelengths, demonstrating the successful routing of incident light to the corresponding subpixels without leakage to neighboring pixels. This study outlines the development of a color router with minimal interpixel crosstalk employing an unfocused Gaussian beam as the incident wave. This design paradigm can be seamlessly integrated with interlayer-based crosstalk control methods, showcasing superior interpixel crosstalk mitigation effects. Subsequently, in the following section, we propose a color router with minimal interpixel crosstalk by combining both approaches in the design of a 3D structured color router.

## 4 Interpixel crosstalk-minimized color routers in 3D

**Figure 10:**
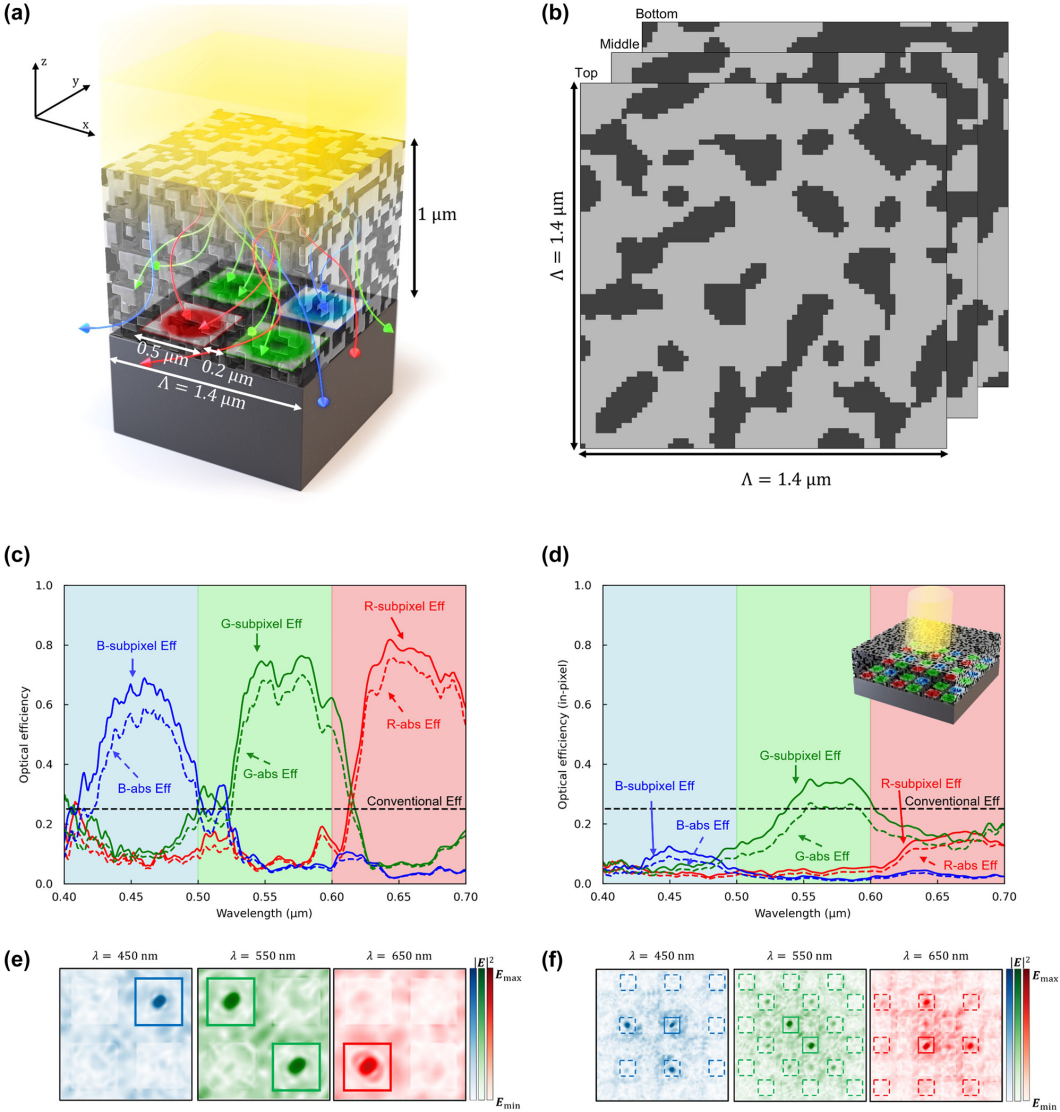
Adjoint optimization of the 3D color router with periodic boundary condition. (a) The 3D optimized color routing-based CMOS image sensor with Bayer pattern. The design region has 1.4 μm periodicity and 1 μm height, giving 245,000 design parameters when the grid spacing is 20 nm. (b) The horizontal cross section of the top, middle, and bottom part of the optimized design region, comprising SiO_2_ (light gray) and TiO_2_ (dark gray), exhibits a symmetric design. (c) The optical efficiency of the subpixels at the visible wavelengths in periodic boundary conditions. The curves indicate transmission normalized optical efficiency, while dashed curves indicate absolute optical efficiency. The dashed lines indicate the efficiency limits of the color-filter based conventional image sensors. (d) The optical efficiency of the subpixels at the central pixel is evaluated across the visible wavelengths, with the optimized design region arranged in a 3 × 3 configuration, as depicted in the inset figure. The curves indicate transmission normalized optical efficiency, while dashed curves indicate absolute optical efficiency. The overall optical efficiencies have dropped significantly due to the interpixel crosstalk. (e) Intensity profiles of the optimized structure at three representative wavelengths (450, 550, 650 nm) in periodic boundary conditions. (f) Intensity profiles of the 3 × 3 configuration of the optimized structure at three representative wavelengths (450, 550, 650 nm). The incident power is leaked to adjacent pixels, indicating significant interpixel crosstalk.

In the previous section, we demonstrated the effectiveness of our proposed methodology in mitigating interpixel crosstalk within color routers through 2D optimization. Here, we extend our investigation to the design of a color router using adjoint optimization within a 3D simulation environment. We then quantitatively assess the interpixel crosstalk exhibited by the resultant structure through measurements of in-pixel optical efficiency. As shown in Figure 10(a), the photodetector arrangement in the 3D configuration consists of a periodic array comprising one red (R), two green (G), and one blue (B) subpixels, which forms the Bayer pattern [1], [61]. This arrangement aligns the polarization symmetry of the incident plane wave – comprised of equal magnitudes of *x*- and *y*-polarization components – with the layout of the photodetectors, thereby ensuring that the performance of the three-dimensional color router is robust against variations in the incident wave’s polarization. For example, the optimized design shows a mirror symmetry at *y* = *x* axis. The *x*-polarized plane wave may show the same result compared to the *y*-polarized plane wave.

The photodetectors are square shaped with a side length of 0.5 μm and separated by a DTI region of 0.2 μm in width. The design space extends 1 μm in the *z*-axis direction and spans 1.4 μm in both the *x*- and *y*-axis directions. Notably, compared to the 2D counterpart, the height of the design area is halved from 2 μm to 1 μm, and the width is reduced by 0.1 μm from 1.5 μm to 1.4 μm. Although reducing the height of the design area might decrease the potential efficiency of the optimized color router, the simplification of the design may reduce the fabrication complexity in the realization. This is substantiated by the arrangement of photodetectors within the design area – three photodetectors are placed within a 1.5 μm width in the two-dimensional simulation compared to two within a 1.4 μm width in the three-dimensional model.

Color routing within a 3D spatial context presents an intriguing challenge that surpasses the complexity of its 2D counterpart. This increased complexity arises from the necessary adjustments to the *k*_*x*_ and *k*_*y*_ components of the incident wave. Despite these increased complexities and the significant expansion of design parameters and spatial considerations in three dimensions, the effectiveness of the optimization approach is anticipated to rival that of the two-dimensional model through appropriate optimization strategies. To manage the computational demands, methodologies such as PML or Bloch boundary conditions are employed on a single unit cell to alleviate computational burdens.

We defined the FOM of the adjoint optimization as maximizing the intensity of the electric field at the center of each subpixel. Due to the Bayer pattern arrangement of the photodetector array, the intensities for the two green subpixels were aggregated and then averaged to ensure equitable optimization across each color. The flux to the green subpixels was normalized to be identical, reflecting the symmetry in the incident wave and the design space. Our FOM utilized for the three-dimensional (3D) optimization is defined as

$$\begin{array}{ll} & F=\alpha \int {\left|E\left(x_{R},\lambda \right)\right|}^{2}+\beta \int \frac{{\left|E\left(x_{G_{1}},\lambda \right)\right|}^{2}+{\left|E\left(x_{G_{2}},\lambda \right)\right|}^{2}}{2}\\ & \;+\gamma \int {\left|E\left(x_{B},\lambda \right)\right|}^{2} \end{array}$$

where $x_{R},x_{G_{1}},x_{G_{2}},x_{B}$ indicate the centers of each subpixel, and ***E*** is the electric field. The visible spectrum was segmented into three distinct regions: 400–500 nm, 500–600 nm, and 600–700 nm, corresponding to red (R), green (G), and blue (B) colors, respectively. The field intensities were summed at uniformly spaced wavelengths to evaluate the average efficiency within each wavelength band. The coefficients *α*, *β*, and *γ* are normalization factors to correct for intensity variations across different wavelengths. To ensure uniform illumination for the two green subpixels, we adjusted the design area to achieve diagonal mirror symmetry, enabling equal light absorption across symmetrical green subpixels. Given the computational constraints associated with 3D simulations, we established a grid spacing of 20 nm, resulting in 245,000 design parameters encompassing the entire design area.

In accordance with prior research, we utilized adjoint optimization under the assumption of Bloch boundary conditions to design a color router. This approach was then followed by a quantitative analysis of the router’s optical efficiency and the degree of interpixel crosstalk observed. As observed in previous optimal designs of 2D color routers employing Bloch boundary conditions and plane waves, this approach was anticipated to yield increased optical efficiency, albeit possibly at the cost of increased interpixel crosstalk.

We selected materials (SiO_2_, TiO_2_) that are lossless within the visible spectrum yet exhibit significant dielectric contrast [62], potentially increasing polarizability and thus enhancing color routing efficiency. The dielectric constants are assumed to be 7.0 for TiO_2_, and 11.8 for the photodetector across the visible spectrum. These materials were represented as densities within the design, with SiO_2_ set as *ρ* = 0 and TiO_2_ as *ρ* = 1, with the initial structure configured to a material density between 0.45 and 0.55. Following the methodology employed in prior 2D adjoint optimization, the structure yields a color router comprising SiO_2_ and TiO_2_, as shown in Figure 10(a). Figure 10(b) displays a horizontal cross section of a color router featuring diagonal mirror symmetry, with 50 layers stacked vertically to form a multi-layer configuration. The optimized color router exhibits outstanding color routing capabilities, attaining an average transmission normalized optical efficiency exceeding 70 %, as shown in Figure 10(c). The average optical efficiencies for red, green, and blue wavelengths were 62.0 %, 56.0 %, and 49.1 %, respectively, with corresponding peak efficiencies of 81.8 %, 76.3 %, and 68.9 %.

Figure 10(e) shows the field intensity profile at three representative wavelengths (450, 550, 650 nm) calculated at the surface of the photodetector, illustrating the effective wavelength-dependent routing to the corresponding subpixels. The symmetrical structure ensures equal intensity distribution across the two green subpixels, a crucial factor in assessing CMOS image sensor performance. As shown in Figure 10(d) inset, we constructed a supercell comprising nine unit cells of the optimally designed color router arranged in a 3 × 3 configuration to analyze the interpixel crosstalk of the optimized structure quantitatively. We then positioned a PML at the end of the *x*–*y* axis and computed the in-pixel optical efficiency using an unfocused Gaussian beam source. Consistent with predictions from 2D color router design results, in-pixel optical efficiency for the red and blue subpixels falls below 20 %, indicative of severe interpixel crosstalk, as shown in Figure 10(d). Figure 10(f) further illustrates the field intensity profile at three representative wavelengths, revealing the undesired routing of incident waves to the subpixels of neighboring pixels. This observation underscores the significant disparity between the overall optical efficiency and the in-pixel optical efficiency in 3D color routers designed using a combination of Bloch boundary conditions and plane waves.

**Figure 11:**
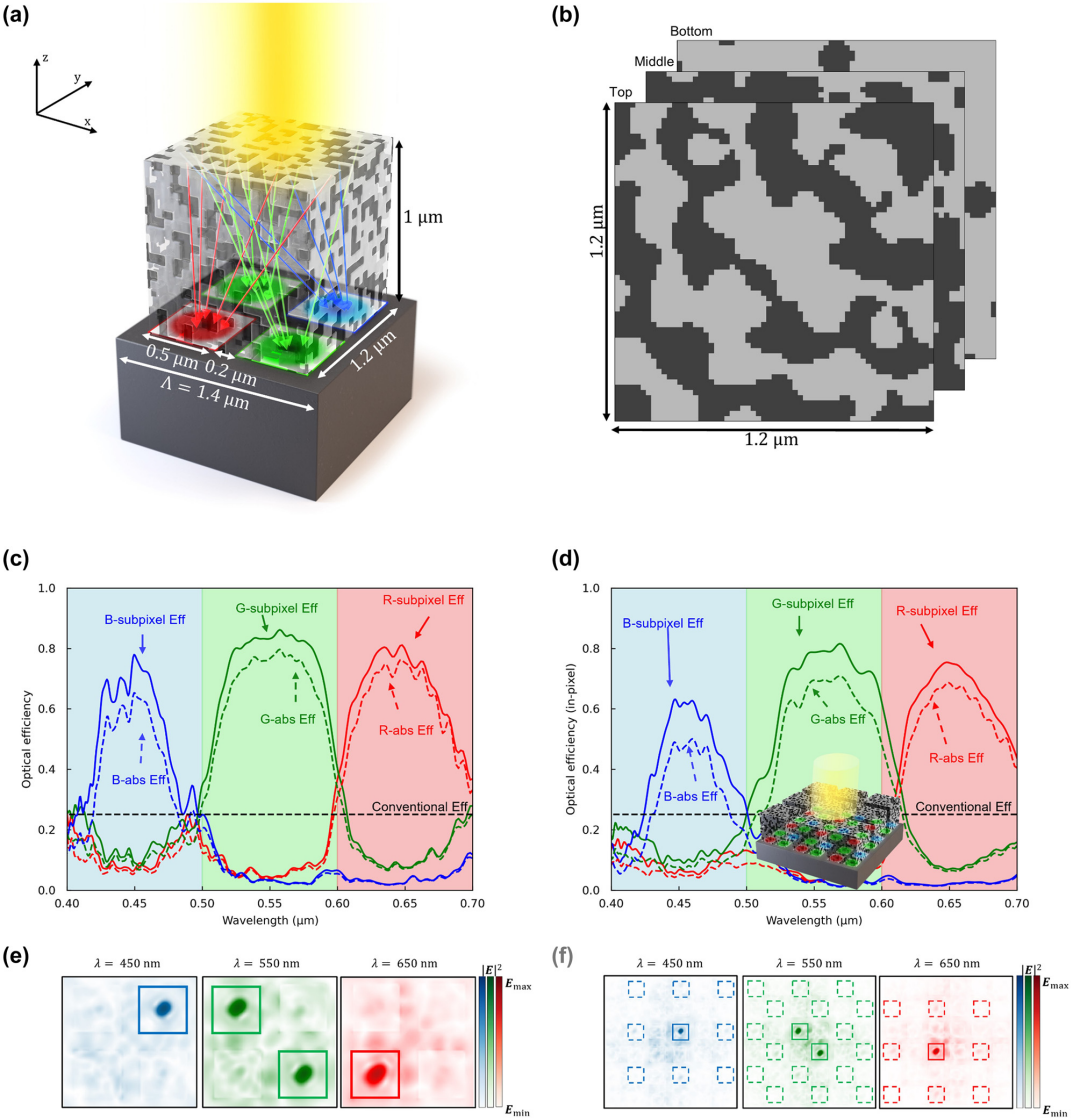
Interpixel crosstalk-minimized color router in 3D. (a) The 3D interpixel crosstalk-minimized CMOS image sensor with Bayer pattern. The design region includes 0.1 μm air gaps on both sides, with 1.4 μm periodicity and 1 μm height, giving 180,000 design parameters when the grid spacing is 20 nm. (b) The horizontal cross section of the top, middle, and bottom part of the optimized design region, comprising SiO_2_ (light gray) and TiO_2_ (dark gray), exhibits a symmetric design. (c) The optical efficiency of the subpixels at the visible wavelengths in periodic boundary conditions. The curves indicate transmission normalized optical efficiency, while dashed curves indicate absolute optical efficiency. The dashed lines indicate the efficiency limits of the color-filter based conventional image sensors. (d) The optical efficiency of the subpixels at the central pixel is evaluated across the visible wavelengths, with the optimized design region arranged in a 3 × 3 configuration, as depicted in the inset figure. The curves indicate transmission normalized optical efficiency, while dashed curves indicate absolute optical efficiency. The optical efficiency remains relatively well maintained, indicating effective suppression of interpixel crosstalk. (e) Intensity profiles of the optimized structure at three representative wavelengths (450, 550, 650 nm) in periodic boundary conditions. (f) Intensity profiles of the 3 × 3 configuration of the optimized structure at three representative wavelengths (450, 550, 650 nm). The incident power is effectively confined to the center pixel, demonstrating successful mitigation of interpixel crosstalk.

The methodologies employed to mitigate interpixel crosstalk in the 2D color router design, such as the incorporation of (1) an air gap interlayer and (2) a customized incident source, were simultaneously applied to enhance the performance of the 3D color router. As depicted in Figure 11(a), the design region possesses dimensions of 1.2 μm in both the *x* and *y* axes, with a height of 1 μm along the *z*-axis. Additionally, we placed a 0.2 μm width air gap at the end of the design area along the *x* and *y* directions. The constituent materials within the design area comprise SiO_2_ and TiO_2_. An unfocused Gaussian beam with a diameter of 1.4 μm was directed onto these structures, with PML employed to define the boundary of the air gap. Figure 11(b) presents a horizontal cross-sectional view of the optimized color router, revealing a diagonal mirror symmetry as depicted in Figure 10(b). Figure 11(c) details the optical efficiency computed by applying incident plane wave to the optimized structure under Bloch boundary conditions, achieving an impressive peak transmission-normalized optical efficiency exceeding 70 % in blue subpixels, and 80 % in red and green subpixels. This performance significantly exceeds that depicted in Figure 10(c), indicating the superior capabilities of the proposed color router compared to conventional designs, even without consideration for interpixel crosstalk. Figure 11(e) depicts the field intensity profile at three distinct wavelengths (450, 550, 650 nm), computed at the surface of the photodetector, which demonstrates precise guidance of the incident wave to individual subpixels without discernible color crosstalk. Additionally, to quantitatively assess the interpixel crosstalk of the proposed design, we arranged the unit cells of the optimized color router in a 3 by 3 configuration, and an unfocused Gaussian beam was employed to measure the in-pixel optical efficiency, as depicted in the inset figure of Figure 11(d). As indicated in Figure 11(d), the blue subpixel exhibits a peak in-pixel optical efficiency exceeding 60 %, while the red and green subpixels demonstrate peaks exceeding 70 %, marking a substantial improvement over the corresponding values depicted in Figure 10(d). Specifically, the average optical efficiencies for red, green, and blue wavelengths were 58.6 %, 64.5 %, and 38.8 %, respectively, with peak efficiencies recorded at 75.4 %, 81.5 %, and 63.1 %, respectively. These results signify a notable increase in in-pixel optical efficiency by 58.0 %, 46.4 %, and 50.7 %, respectively, compared to color routers devoid of interlayer and optimized solely for plane waves. Moreover, Figure 11(f) illustrates the field intensity profile at the central wavelength for each color, suppressing the fraction entering the subpixels of adjacent unit cells to levels below 1 %. Although the pixel size used in this study deviates significantly from that of commercially available CMOS image sensors, the potential for designing color routers with enhanced efficiency exists by increasing pixel size and expanding the design area. Nevertheless, further investigations are necessary to develop methods for reducing costs in multi-layer fine patterning processes and minimizing alignment errors between layers, which would facilitate the commercialization of CMOS image sensors based on color routing.

## 5 Conclusions

In this paper, we present the first assessment of interpixel crosstalk in color routers designed for CMOS image sensors, illustrating the significant interpixel crosstalk observed in previously investigated color routers. To mitigate the issue, we introduce two innovative approaches: (1) the incorporation of a physical interlayer and (2) the utilization of a customized incident wave. We considered various candidates for physical interlayers, including tungsten and air, with tungsten showing excellent control of interpixel crosstalk in our designs, albeit with associated absorption and reflection losses. Conversely, the use of an air gap significantly reduced these losses, though it did not entirely eliminate interpixel crosstalk. Consequently, we advocate for an optimal design approach leveraging a customized source to effectively regulate interpixel crosstalk, thereby obviating the need for an interlayer process. The color router optimized through this method exhibited a reduction in interpixel crosstalk by a factor of 7 compared to conventionally designed counterparts.

Furthermore, we introduced our approach to 3D color routing that combines interlayer and customized source methods. This innovative method enhances color routing efficiency by effectively managing interpixel crosstalk. Our proposed approach has successfully mitigated interpixel crosstalk, achieving 75.4 %, 81.5 %, and 63.1 % peak in-pixel optical efficiency for red, green, and blue colors, respectively. Compared to the optimized 3D color router utilizing Bloch boundary conditions and plane wave, our method has resulted in a remarkable reduction of interpixel crosstalk by up to 30 %, along with substantial improvements of 58.0 %, 46.4 %, and 50.7 % in in-pixel optical efficiency for red, green, and blue, respectively.

The color router proposed in this study is anticipated to facilitate the commercialization of color routing-based CMOS image sensors. However, the realization of the proposed color routers may have several challenges in the fabrication cost and the resolution of the lithography technique. Advances in nanolithography have already demonstrated the ability to achieve feature sizes as small as 10–20 nm. However, the cost of using high-resolution lithography is highly expense. In addition, a multi-layer structure shown in this work may require substantial fabrication costs. Therefore, finding an optimum point between color routing efficiency and lithography resolution (e.g., fabrication cost) might be necessary. Additionally, a color router-based CMOS image sensor may require angle-independent performance at the desired incidence angles.

## Figures and Tables

**Figure A1:**
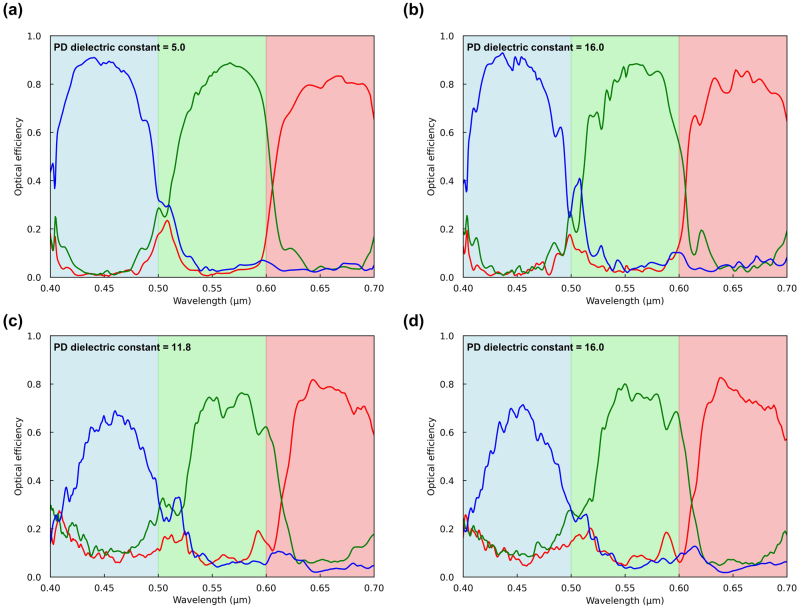
Transmission-normalized optical efficiency of the subpixels at the visible wavelengths in periodic boundary conditions. (a) The dielectric constant of the 2D photodetector is assumed as 5.0. (b) The dielectric constant of the 2D photodetector is assumed as 16.0. (c) The dielectric constant of the 3D photodetector is assumed as 11.8. (d) The dielectric constant of the 3D photodetector is assumed as 16.0.

**Figure A2:**
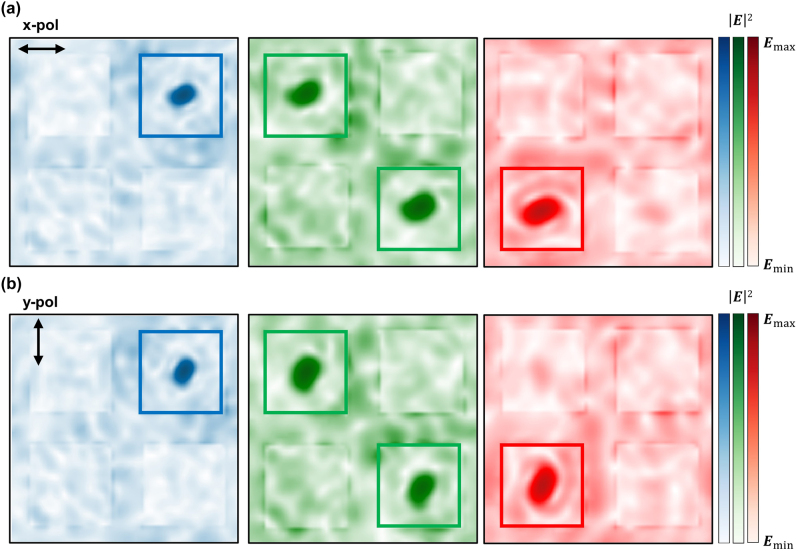
Intensity profiles of the optimized structure at three representative wavelengths in periodic boundary conditions. (a) Intensity profile with *x*-polarization plane wave. (b) Intensity profile with *y*-polarization plane wave.

## Appendix A: Dielectric constants of the photodetectors

We selected 5.0 (2D) and 11.8 (3D) dielectric constants for the photodetectors to reduce computational load. Using low dielectric constants significantly reduces the simulation time. For instance, the refractive index of Si (*n* ≃ 4.0) may require a grid spacing of 5 nm in order to satisfy a minimum grid space of *λ*/(20 ⋅ *n*). It substantially increases the simulation time. In our computational resource (96-core AMD Ryzen Threadripper Pro 7995WX), optimizing a 3D color router with a minimum grid spacing of 5 nm requires approximately 900 h. Therefore, we chose a lower dielectric constant of the photodetector in the optimization and then validated the optimized result with a higher electric constant (16.0), as illustrated in Figure A1. These results indicate that our color router design maintains efficiency even with photodetectors of higher dielectric constants.

## Appendix B: Polarization insensitivity of the optimized design

We designed the structure to be symmetric, ensuring that the two green pixels have uniform efficiency regardless of polarization. The source, design, and photodetector are symmetric across the *y* = *x* axis. Therefore, simulation results using either an *x*-polarized or *y*-polarized plane wave should be identical, as shown in Figure A2.
